# Artificial intelligence to predict in-hospital mortality using novel anatomical injury score

**DOI:** 10.1038/s41598-021-03024-1

**Published:** 2021-12-07

**Authors:** Wu Seong Kang, Heewon Chung, Hoon Ko, Nan Yeol Kim, Do Wan Kim, Jayun Cho, Hongjin Shim, Jin Goo Kim, Ji Young Jang, Kyung Won Kim, Jinseok Lee

**Affiliations:** 1 Department of Trauma Surgery, Jeju Regional Trauma Center, Cheju Halla General Hospital, Jeju, Republic of Korea; 2 Department of Biomedical Engineering, Kyung Hee University, Yongin, Republic of Korea; 3 Department of Thoracic and Cardiovascular Surgery, Chonnam National University Hospital and Chonnam National University Medical School, Gwangju, Republic of Korea; 4 Department of Trauma Surgery, Gachon University Gil Medical Center, Incheon, Republic of Korea; 5 Wonju Trauma Center, Yonsei University Wonju College of Medicine, Wonju, Republic of Korea; 6 Trauma Center, Wonkwang University Hospital, Iksan, Republic of Korea; 7 Department of Surgery, National Health Insurance Service, Ilsan Hospital, Goyang, Republic of Korea; 8 Department of Radiology, Asan Image Metrics, Asan Medical Center, University of Ulsan College of Medicine, Seoul, Republic of Korea

**Keywords:** Risk factors, Biomedical engineering

## Abstract

The aim of the study is to develop artificial intelligence (AI) algorithm based on a deep learning model to predict mortality using abbreviate injury score (AIS). The performance of the conventional anatomic injury severity score (ISS) system in predicting in-hospital mortality is still limited. AIS data of 42,933 patients registered in the Korean trauma data bank from four Korean regional trauma centers were enrolled. After excluding patients who were younger than 19 years old and those who died within six hours from arrival, we included 37,762 patients, of which 36,493 (96.6%) survived and 1269 (3.4%) deceased. To enhance the AI model performance, we reduced the AIS codes to 46 input values by organizing them according to the organ location (Region-46). The total AIS and six categories of the anatomic region in the ISS system (Region-6) were used to compare the input features. The AI models were compared with the conventional ISS and new ISS (NISS) systems. We evaluated the performance pertaining to the 12 combinations of the features and models. The highest accuracy (85.05%) corresponded to Region-46 with DNN, followed by that of Region-6 with DNN (83.62%), AIS with DNN (81.27%), ISS-16 (80.50%), NISS-16 (79.18%), NISS-25 (77.09%), and ISS-25 (70.82%). The highest AUROC (0.9084) corresponded to Region-46 with DNN, followed by that of Region-6 with DNN (0.9013), AIS with DNN (0.8819), ISS (0.8709), and NISS (0.8681). The proposed deep learning scheme with feature combination exhibited high accuracy metrics such as the balanced accuracy and AUROC than the conventional ISS and NISS systems. We expect that our trial would be a cornerstone of more complex combination model.

## Introduction

Traumatic injuries have long been scaled to assess the severity of patients and especially to predict mortality. To quantify the severity of such injuries, various metrics using anatomic and physiologic elements have been established based on conventional statistical tools such as logistic regression (LR)^1–7^. Since the 1970s, the injury severities have been classified through an anatomical-based coding system named the abbreviated injury scale (AIS). The AIS classifies individual injuries by body region from one to six. The system has been constantly revised and improved, and the latest revision (AIS 2005—Update 2008 and AIS 2015), including 1999 injury descriptors, was recently announced^1^.

The most popular model to assess the trauma severity is the injury severity score (ISS), which pertains to the sum of the squares of the three highest AIS scores^2^. Similarly, the new ISS (NISS), which is designated to replace the ISS, pertains to the squares of the three highest AIS scores regardless of the body region^3–5^. Using the ISS and NISS, mortality has been predicted based on a certain score; however, the associated mortality prediction accuracy is low for patients with multiple injuries in different areas^6,7^.

As a more complex combination model including the physiological status, the trauma and ISS (TRISS) has been widely used^8^. In addition, other combination models, such as A Severity Characterization of Trauma (ASCOT)^9^, the Harborview Assessment for Risk of Mortality (HARM)^10^, the Trauma Risk Adjustment Model (TRAM)^11^, and the Kampala Trauma Score (KTS)^12^ were developed. However, these scoring systems are overly complex to be applied in actual practice. Thus, the anatomic injury scoring systems based on ISS and NISS are still widely used despite their low prediction accuracy, and it is necessary to develop a simple and accurate prediction method.

Recently, artificial intelligence (AI) technologies have been applied in various medical domains to predict the patients’ outcome with a high accuracy^13–15^. However, in the trauma field, an effective AI prediction model has not been established yet. Therefore, this study was aimed at developing an AI model to accurately predict mortality as an alternative to ISS and NISS.

## Methods

### Patients and datasets

This study was approved by the four institutional review boards of Wonkwang University Hospital (WKUH), Chonnam National University Hospital (CNUH), Wonju Severance Christian Hospital (WSCH), and Gachon University Gil Hospital (GUGH). Informed consent was waived. All methods were performed in accordance with the relevant guidelines and regulations. These four hospitals include the regional trauma centers of South Korea, corresponding to the level-1 trauma centers in the United States. In each regional trauma center, three or more dedicated trauma coordinators and surgeons review the medical records of the trauma patients to be input to the Korean trauma data bank (KTDB). To be registered in the KTDB as a trauma patient, the following eligibility criteria must be satisfied: (1) discharged with hopelessness, died in the trauma bay, or transferred to other hospitals, and (2) admitted in a trauma center^16^. However, among the patients satisfying the above conditions, patients with only a superficial injury, first-degree burn and corrosive injury, frostbite, or intoxications are not registered in the KTDB. For this study, we considered the following inclusion criteria: (1) trauma patients registered in the KTDB, (2) adult patients aged over 19, (3) patients who stayed more than 6 h in the trauma center.

In the KTDB, the AIS 2005—Update 2008 data of 42,933 patients^17^ registered in four trauma centers (WKUH [from Jan 2017 to Dec 2019], CNUH [from Jan 2014 to Dec 2019], WSCH [from Jan 2014 to Dec 2019], and GUGH [from Jan 2014 to Dec 2019]) are available. We excluded the data of patients aged less than 19 (*n* = 4562) or those who spent less than 6 h in the hospital (*n* = 648). The patient information, including age, gender and the AIS scores, is summarized in Table 1. Among the included 37,762 patients, 36,493 (96.6%) survived (average age: 57.0 ± 17.80) and 1269 (3.4%) deceased (average age: 64.66 ± 16.42). More details of the age and gender according to the four trauma centers are summarized in Supplementary Table 1.

**Table 1:** Patient information of age, gender, and the AIS scores (0–6) for six body regions: head/neck, face, thorax, abdomen, extremity, and external, according to the survived and deceased groups.

Characteristics	Total	Survived	Deceased	*p*-value
	*n* = 37,762	*n* = 36,493 (96.6%)	*n* = 1269 (3.4%)	
Age	57.26 ± 17.81	57.00 ± 17.80	64.65 ± 16.42	< 0.001
Sex				
Male	24,734	23,868 (96.5%)	866 (3.5%)	< 0.001
Female	13,028	12,625 (96.9%)	403 (3.1%)	
AIS (head/neck)				
0	25,810	25,454	356	< 0.001
1	1282	1280	2	
2	3369	3312	57	
3	3589	3439	150	
4	1970	1713	257	
5	1721	1274	447	
6	21	21	0	
AIS (face)				
0	32,344	31,294	1050	< 0.001
1	2480	2407	73	
2	2790	2678	112	
3	132	106	26	
4	16	8	8	
5	0	0	0	
6	0	0	0	
AIS (thorax)				
0	29,421	28,634	787	< 0.001
1	581	564	17	
2	2325	2242	83	
3	4760	4475	285	
4	565	484	81	
5	109	94	15	
6	1	0	1	
AIS (abdomen)				
0	31,640	30,720	920	< 0.001
1	369	365	4	
2	3421	3266	155	
3	1690	1603	87	
4	598	515	83	
5	44	24	20	
6	0	0	0	
AIS (extremity)				
0	18,262	17,478	784	< 0.001
1	2707	2702	5	
2	10,337	10,126	211	
3	5951	5765	186	
4	384	349	35	
5	121	73	48	
6	0	0	0	
AIS (external)				
0	25,448	24,572	876	0.1407
1	11,345	10,988	357	
2	795	772	23	
3	82	74	8	
4	28	27	1	
5	55	52	3	
6	9	8	1	

**Figure 1:**
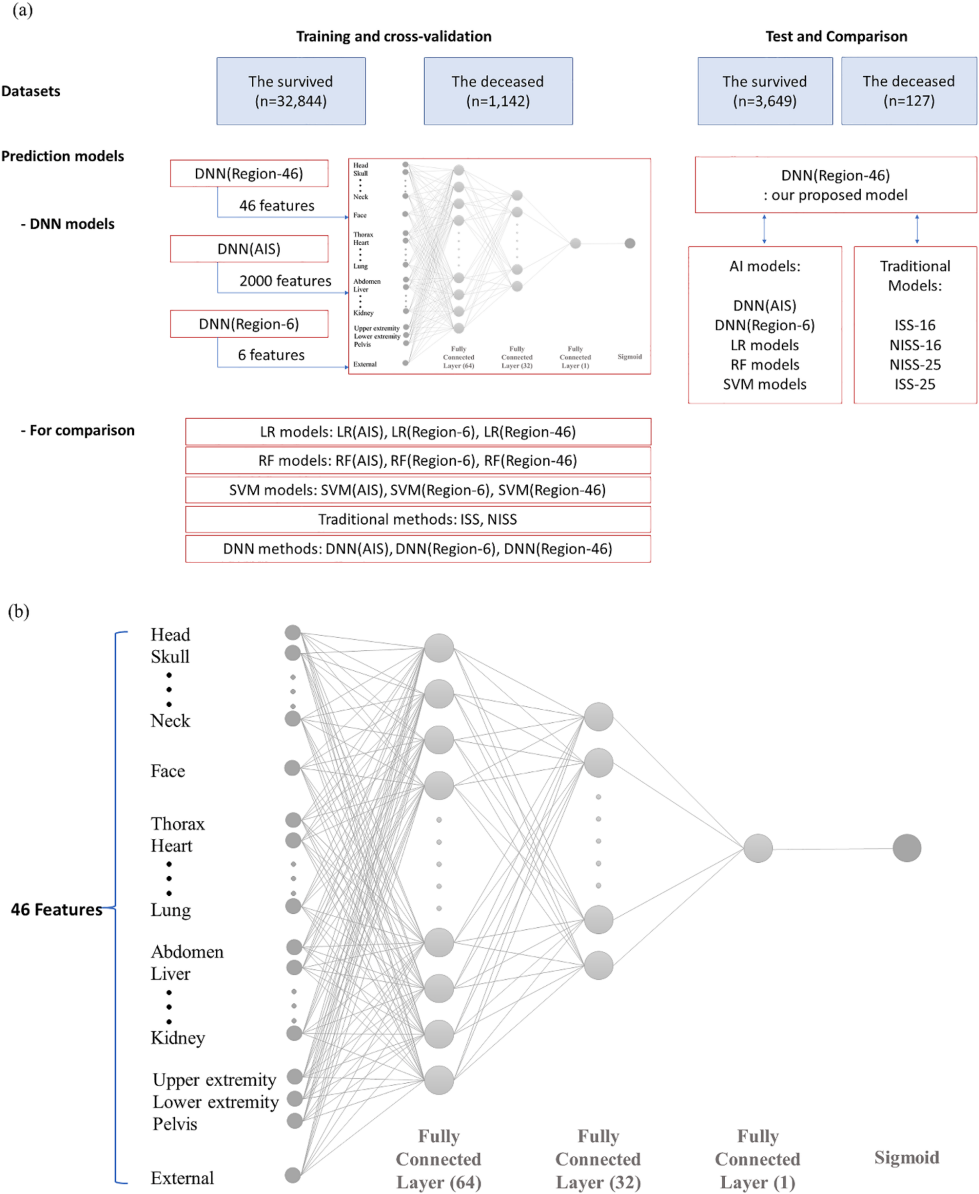
Overview of training and validation of the AI models to predict the in-hospital mortality in trauma patients: (**a**) Process flow of the AI model development, and (**b**) DNN (Region-46) architecture with a five-layer deep neural network consisting of an input layer, three fully connected (FC) layers, and output layer.

The data of 37,762 patients were considered and divided into the training and testing cohort in a 9:1 ratio in a stratified fashion according to the trauma center and survival status. As presented in Table 2 and Fig. 1a, the training and testing datasets included 33,986 (32,844 survived and 1142 deceased) and 3776 (3649 survived and 127 deceased) patients, respectively. The testing dataset was used only to evaluate the performance.

**Table 2:** Summary of training, validation, and testing datasets.

Data source	Group	Training	Testing	Total
WKUH	Survival	3722	413	4135
	Mortality	159	18	177
JNUH	Survival	8827	981	9808
	Mortality	394	44	438
WSCH	Survival	12,278	1364	13,642
	Mortality	328	36	364
GUGH	Survival	8017	891	8908
	Mortality	261	29	290
Total	Survival	32,844	3649	36,493
	Mortality	1142	127	1269
	Total	33,986	3776	37,762

### New trauma severity features

The original AIS codes have 1999 injury codes, which are excessively complex to be used as input features in AI models and may lead to model overfitting. Consequently, we established new anatomical categories named Region-46 and Region-6 by reducing the AIS codes into 46 categories and six categories, respectively, based on the organ locations.

To generate Region-46, we reduced the 1999 AIS codes to 46 features through organization according to the organ location, clinical significance, and frequency. The complete list of Region-46 is presented in Supplementary Table 1. In the head region, we categorized the codes by the cerebellum, cerebellum epidural, intracerebellar, cerebellum subdural, cerebrum, cerebrum epidural, intracerebral, cerebrum subdural, skull, and head. The relatively less important and less frequent injuries were categorized as head or skull. In the face region, no frequently occurring code represented a severe injury, and thus, all the corresponding codes were categorized as the face. In the neck region, we categorized the codes by the neck vessel, neck esophagus, neck trachea, and cervical spine. The other corresponding codes were categorized as the neck. In the thorax region, we categorized the codes by the thorax vessel, bronchus, diaphragm, thorax esophagus, heart, lung, pericardium, thorax trachea, rib, and thoracic spine. The other codes were categorized as the thorax. In the abdomen region, we categorized the codes by the abdomen vessel, bladder, colon, duodenum, small bowel, kidney, liver, mesentery, omentum, pancreas, rectum, spleen, stomach, and lumbar spine. The other codes were categorized as the abdomen. In the upper extremity region, all the codes were categorized as upper extremity because the scores of all the codes were low overall. In the lower extremity region, the pelvis was the most clinically significant organ, and the other injuries were less significant. Thus, we categorized the codes as the pelvis and lower extremity. In the external region, the AIS scoring codes were low, and thus, all the codes were categorized as external. In Supplementary Table 2, we also presented the example of how AIS codes were converted to Region-46 codes for a novice coder with trauma knowledge to understand the process.

When using the Region-46 categories as input features, each input feature was the sum of the squares of the AIS scores for the same feature. For instance, if two AIS scores (valued 2 and 3) corresponded to the neck among the Region-46, the AI model input for the neck feature was $2^{2} + 3^{2} = 13$. In this manner, each patient data point pertained to 46 feature values used for the AI model input layer to predict mortality.

Region-6 was considerably simpler than Region-46, as it scored the body regions identical to the six regions of the ISS (head/neck, face, thorax, abdomen, extremity, and external). ISS takes the highest AIS severity code in each of the three most severely injured body regions. Each three AIS score is squared and added (i.e. ISS = 5^2^ + 4^2^ + 2^2^).

### Training and cross-validation of the DNN models

To develop the AI models for mortality prediction, a five-layer DNN was adopted. We developed three DNN models with variable input features, as follows: (1) DNN (Region-46) with 46 input features, (2) DNN (AIS) with the original 1999 AIS codes, and (3) DNN (Region-6) with six body regions.

Furthermore, up to five hidden layers were examined, along with each layer depth (node) up to the previous layer depth (node). For the fully connected layers (FCs), the dropout rate was varied from 0 to 0.5 in 0.1 increments. The last FC layer was fed to a sigmoid layer, providing the probabilities for the patient mortality. We implemented and trained the models using TensorFlow and Keras. Moreover, model training was realized using the ADAM optimizer and a binary cross-entropy cost function with a learning rate of 0.0001 and batch size of 64 on an NVIDIA GeForce GTX 1080 Ti GPU.

For the hyperparameter search and cross-validation, a grid search with a 10-repeated tenfold cross-validation was performed. Among the 10 folds, a single fold was retained as the validation data, and the remaining nine folds were used as the training data. Specifically, the training data (*n* = 33,986) were randomly shuffled for each trauma center and partitioned into 10 equal folds in a stratified manner: Each fold included 3284 survived and 114 deceased data. The process was repeated 10 times, with each of the 10 folds used once as the validation data. Because the amount of survived data overwhelmed the deceased data, we randomly downsampled the survived data. By matching the amount of data in the two groups, the model bias toward survived data could be minimized.

Considering the cross-validation analysis results, the five-layer DNN was selected. The architecture of DNN (Region-46) is illustrated in Fig. 1b. The proposed five-layer DNN included an input layer, three FC layers, and an output layer. The input layer containing 46 features was fed to three FC layers in series, consisting of 64, 32, and 1 node(s) with a leaky rectified linear unit. To alleviate the overfitting issue, dropout and L2 regularization were applied in the last two FC layers. For the second and third FC layers, the dropout rates were 0.5 and 0.3, respectively, and regularization parameters were 0.001. The last FC layer was fed to a sigmoid layer.

By repeating this stratified tenfold cross-validation process 10 times, 100 models from the five-layer DNN were derived. Based on these models, we ensembled the results by averaging the final outputs associated with the mortality probabilities.

### Performance evaluation of AI models

The prediction performance of the DNN models was evaluated using the isolated testing dataset (*n* = 3776) and by applying the sensitivity, specificity, accuracy, and balanced accuracy metrics, defined as

1 $$Sensitivity = { }\frac{TP}{{TP + FN }},$$

2 $$Specificity = { }\frac{TN}{{TN + FP }},$$

3 $$Accuracy = { }\frac{TP + TN}{{TP + TN + FP + FN }},$$

4 $$Balanced \;Accuracy = { }\frac{Sensitivity + Specificity}{{2 }},$$

where TP, TN, FP, and FN represent the true positive, true negative, false positive, and false negative, respectively. The balanced accuracy was used to account for the considerable imbalance between the survived and deceased groups (29:1).

Next, we compared the performance of the proposed DNN model and other external AI models such as LR, random forest (RF), and support vector machine (SVM). The models were separately trained to perform cross-validation and determine the optimal hyperparameters.

Finally, we compared the results from the conventional ISS and NISS techniques, as follows:

1. ISS-16: mortality declared if ${\text{ISS}} \ge 16$
2. ISS-25: mortality declared if ${\text{ISS}} \ge 25$
3. NISS-16: mortality declared if ${\text{NISS}} \ge 16$
4. NISS-25: mortality declared if ${\text{NISS}} \ge 25$

## Results

For all 37,762 patients, we counted the number of patients associated with each of the Region-46 features and summarized the counted numbers in Supplementary Table 3. The number of patients associated with lower extremity (*n* = 12,280) was the highest, followed by upper extremity (*n* = 11,548), face (*n* = 8310), head (*n* = 7200), rib (*n* = 6147) and thorax (*n* = 4763). Note that each patient may belong to multiple features due to multiple injuries.

### Training and cross-validation of the DNN models

The results of the cross-validation accuracy with the metrics of sensitivity, specificity, accuracy and balanced accuracy are presented in Supplementary Table 4. The highest balanced accuracy (84.61%) corresponded to the proposed model, DNN (Region-46), followed by that of the DNN (Region-6) (83.32%) and DNN (AIS) (80.64%). DNN (AIS) achieved a smaller balanced accuracy than that of DNN (Region-46) and DNN (Region-6), indicating that overfitting may have occurred, as expected; 1999 input features are too many to learn patterns based on 33,986 training data.

### Validation of DNN models in the testing dataset

The prediction performances of the DNN models evaluated using the independent testing dataset (*n* = 3776) are summarized in Table 3. The highest balanced accuracy (85.05%) corresponded to DNN (Region-46), followed by that of DNN (Region-6) (83.62%) and DNN (AIS) (81.27%). The highest area under receiver operating characteristic (AUROC) corresponded to DNN (Region-46) (0.9084), followed by that of DNN (Region-6) (0.9013) and DNN (AIS) (0.8819). Thus, we chose DNN (Region-46) as the proposed AI model to predict the in-hospital mortality of trauma patients. Notably, DNN (Region-46) demonstrated a high prediction performance in terms of sensitivity (83.46%), specificity (86.63%), and accuracy (86.52%).

**Table 3:** Comparison of the prediction performances of the prediction models on the test dataset.

Model	TN	FP	FN	TP	Sensitivity (%)	Specificity (%)	Accuracy (%)	Balanced accuracy (%)	AUROC
LR (AIS)	3200	449	32	95	74.80	87.70	87.26	81.25	0.8770
RF (AIS)	2720	929	20	107	84.25	74.54	74.87	79.40	0.8598
SVM (AIS)	3032	617	21	106	83.46	83.01	83.10	83.28	0.8943
DNN (AIS)	3230	419	33	94	74.02	88.52	88.03	81.27	0.8819
LR (Region-6)	3059	590	25	102	80.32	83.83	83.72	82.07	0.8819
RF (Region-6)	3090	559	24	103	81.10	84.68	84.56	82.89	0.8867
SVM (Region-6)	3009	640	23	104	81.89	82.46	82.44	82.18	0.8712
DNN (Region-6)	3028	621	20	107	84.25	82.98	83.02	83.62	0.8871
LR (Region-46)	3109	540	24	103	81.10	85.20	85.06	83.15	0.9013
RF (Region-46)	3054	595	23	104	81.89	83.69	83.63	82.79	0.8853
SVM (Region-46)	3091	558	23	104	81.89	84.71	84.61	83.30	0.8829
DNN (Region-46)	3161	488	21	106	83.46	86.63	86.52	85.05	0.9084
ISS-16	2944	705	25	102	80.31	80.68	80.67	80.50	0.8709
ISS-25	3387	262	65	62	48.82	92.82	91.34	70.82	
NISS-16	2618	1031	17	110	86.61	71.75	72.25	79.18	0.8681
NISS-25	3241	408	44	83	65.35	88.82	88.03	77.09	

### Comparison with other prediction models

The prediction performances of the external AI models (LR, RF, and SVM models) and traditional prediction models (ISS-16, ISS-25, NISS-16, and NISS-25) are presented in Table 3. Details of the performance comparison according to the four trauma centers are summarized in Supplementary Table 5.

**Figure 2:**
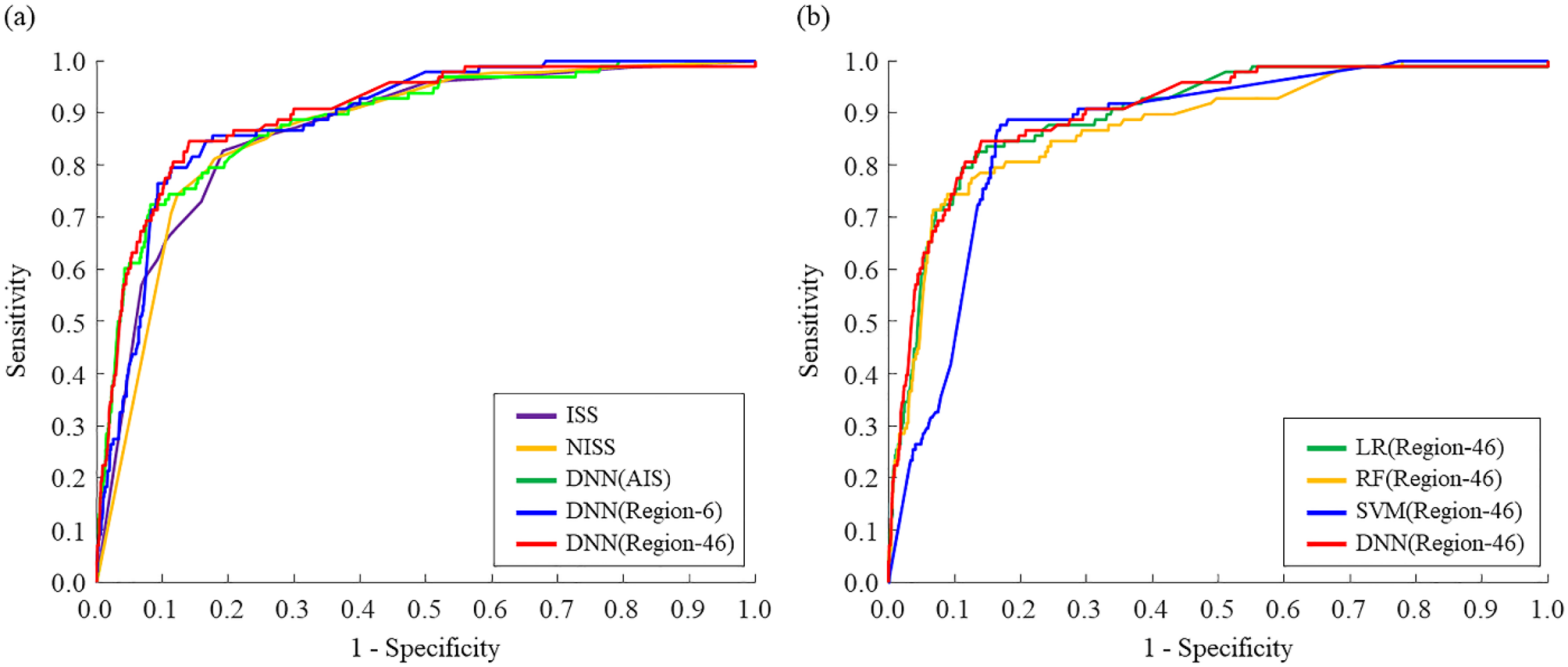
Receiver operating characteristic curves: (**a**) ISS, NISS, DNN (AIS), DNN (Region-6) and DNN (Region-46), and (**b**) four models of logistic regression (LR), random forest (RF), support vector machine (SVM) and DNN based on Region-46: LR (Region-46), RF (Region-46), SVM (Region-46) and DNN (Region-46), respectively.

The highest balanced accuracy pertained to DNN (Region-46) (85.05%) and that of the other AI models ranged from 79.40 to 83.62%. Among the input features based on the DNN, the highest AUROC values pertained to Region-46 (0.9084), followed by those of Region-6 (0.9013), and AIS (0.8819); the corresponding values of ISS and NISS were 0.8709 and 0.8681, respectively (Fig. 2a). Among the different AI models based on Region-46, the highest AUROC corresponded to DNN (Region-46), followed by that of the LR (0.9013), RF (0.8853) and SVM (0.8829) (Fig. 2b).

Notably, the traditional prediction models based on the ISS and NISS exhibited a lower prediction performance (balanced accuracy ranging from 70.82 to 80.50) than AI models based on LR, RF, SVM, and DNN (balanced accuracy ranging from 79.40 to 85.05).

## Discussion

We proposed a DNN model with 46 features, DNN (Region-46) as the AI prediction model to predict the in-hospital mortality of trauma patients, based on a comparison with DNN (AIS), DNN (Region-6), other AI models based on the LR, RF, and SVM methods, and traditional prediction models based on the ISS and NISS. In the independent testing datasets, DNN (Region-46) outperformed the ISS-16 as follows: sensitivity (83.46% vs. 80.32%), specificity (86.63% vs. 80.68%), accuracy (86.52% vs. 80.67%), balanced accuracy (85.05% vs. 80.50%), and AUROC (0.9084 vs. 0.8709).

The AIS-based ISS has been widely used to evaluate the severity of trauma patients since its introduction in the 1970s^1,2,18^. Most subsequent systems such as the NISS^19^, exponential ISS (EISS)^20^, and anatomic profile score (APS)^21^ could not supplant the ISS4. Nevertheless, the ISS system based on the AIS involves several limitations^22,23^. First, a major severe injury may be underestimated because only the single highest score is selected in the same region. Second, the severity may differ if a different combination of injuries has the same ISS. Third, the ISS and mortality do not have a linear association, and a negative relationship may also occur in certain ranges. Finally, the AIS has the same weighting for different organs even though all organs have a different prognosis.

These limitations of the ISS can be alleviated by using AI models^24^. The DNN model can assign different weights to each input data. Thus, we fragmented the conventional ISS system and used the AI methodology to overcome the problem of nonlinearity and weights for each AIS score. In this study, a key challenge was to decide the predictors and features, as the input variables significantly affect the results of machine learning^20^.

A key advantage of DNN (Region-46) is that it was trained with a large training cohort (*n* = 37,762) with highly comprehensive data involving a reasonable number of AIS score data points. The Ministry of Health and Welfare established up to 17 regional trauma centers in Korea (by 2021). All the regional trauma centers were mandated to register severe trauma patients in the KTBD. Numerous variables, including the demographics, pre-hospital and in-hospital information, time factors, clinical characteristics, vital signs, trauma scores, and final outcome information, were recorded. Therefore, the quantity and quality of the KTDB data were satisfactory^25^.

In general, the DNN involves high computational complexity, although it operates more effectively and flexibly than the RF, LR, and SVM, owing to the learning of the nonlinear problem. Nevertheless, owing to the simplified input features, DNN (Region-46) required only 59.52 s for training and 2.19 ms for instance testing, indicating its suitability for practical mortality prediction. Moreover, DNN (Region-6) provided accurate prediction results, requiring only 50.39 s for training and 2.11 ms for instance testing. Thus, if Region-46 is not promptly available, DNN (Region-6) can be used as an alternative method.

The original AIS codes have 1999 injury codes, which are overly complex to be used as input features in AI models and may lead to model overfitting in the current training dataset involving 37,762 patients. The number of features considerably influences the performance of deep learning. The presence of excessively few or many features does not ensure high performance. In general, many data points are required for numerous features. Consequently, we established a new anatomical category, Region-46, by fragmenting the individual AIS codes and aggregating them into 46 anatomic regions. Our proposed Region-46 (46 input variables) could provide more accurate prediction results using more subdivided features than ISS (six input variables). In addition, it minimizes the overfitting issues using reasonably fewer features than AIS (1999 input variables). The better performance of the Region-46-based AI model was possible because 1999 AIS codes were with a sparse matrix, which contains very few non-zero values.

Another advantage of Region-46 is that we derived it from the conventional AIS coding system. It indicates that the Region-46-based AI model enables us to train the model accurately within the existing coding system, without significant changes in the system such as the coding method. Nevertheless, the advantages of Region-46 are based on the assumption that there is no massive data. It is obvious that we can train AI models more accurately using more features from 1999 AIS codes if we have massive data. We postulate that a considerably larger dataset is required to utilize 1999 AIS codes as input features without overfitting. Thus, future research will proceed in the direction of increasing the number of features by collecting more data, and the ultimate goal is to use all 1999 AIS codes.

In addition to the data sample size, this study involves several limitations to be solved. First, this study is retrospective, although the database was collected prospectively. In this study, we performed a retrospective study to investigate the potential of AI models based on our proposed new anatomical injury score. We believe that new prospective studies will be able to minimize the biases and establish the clinical impact of the prediction model. In addition, the prospective study should be able to update AI models in real-time in more regional trauma centers. For future work, we plan to develop a framework that can use real-time data in connection with all the regional trauma centers in Korea, which will improve the model for better generalization. Second, we excluded the patients who died within six hours from arrival because we assumed that the patients were likely not comprehensively evaluated owing to the insufficient time. This aspect may contribute to selection bias. Thus, our future work would include the patients irrespective of the time of death, which may provide a more robust AI model to predict the severity of trauma patients. Third, the input data did not include the physiologic and demographic parameters such as blood pressure, heart rate, mental status, respiration, age, sex, and comorbidities, while likely leading to an inherently limited accuracy. However, we noted the inherent potential of solely AIS code analysis that would be a basis of the future complex models. In particular, this study highlights the substantial scope for improvement in terms of the injury scale. Compared to ISS, more complex combination models such as TRISS^8^, ASCOT^9^, HARM^10^, TRAM^11^, and KTS^12^ use physiologic and demographic parameters to improve the performance. Although these complex combination models have not provided sufficient accuracy to replace the ISS, they have the potential to provide personalized prediction information of trauma severity. Thus, in our future work, we will extend our model to include the physiologic and demographic parameters towards personalized severity prediction. Finally, in this study, we considered mortality as an indicator of the severity of trauma patients as a conventional scoring system focused on mortality. However, regarding survivors, we also should be able to predict other outcomes such as morbidity, prevention effectiveness, or burden of cost. By developing new indicators that predict these outcomes, we could provide guidelines for entire trauma system including heath care provider, agencies, and institutions.

In conclusion, we developed a five-layer DNN model with an input layer involving 46 new trauma severity features (Region-46), which exhibited a higher prediction performance than the conventional ISS and NISS systems. To the best of our knowledge, this study represents the first attempt to employ AI prediction models using anatomic injury severity information. Furthermore, this study emphasizes the need for reinvention of a new injury severity scoring system. We expect that this research can provide reference to incorporate AI techniques in trauma care and improve our understanding of the determinants of injury severity. We believe our endeavor will contribute to reducing the societal burden of injury.

## Supplementary Information

Supplementary Information 1.

Supplementary Information 2.

## Data Availability

Data are available from the corresponding author upon reasonable request. The data are not publicly available due to restrictions from KTDB policy.

## Code availability

Code is available at https://github.com/HeewonChung92/AIS. The code was written in python 3.6 and Tensorflow 2.0. The files named Code_Train.py and Code_Predict.py are for training and testing the model, respectively. The file named Dataset_mini.mat is for the automatic conversion program from AIS codes to Region-46 codes.
