# Supplementary material for: Artificial intelligence to predict in-hospital mortality using novel anatomical injury score
Source: Sci Rep. 2021 Dec 7;11:23534. doi: 10.1038/s41598-021-03024-1 (PMC8651670; doi:10.1038/s41598-021-03024-1)
Supplement: Supplementary file 1 — Supplementary Information 1. [file 41598_2021_3024_MOESM1_ESM.docx]

**Artificial Intelligence to Predict In-Hospital Mortality Using Novel Anatomical Injury Score**

Wu Seong Kang, M.D^1,†^, Heewon Chung, M.S^2,†^, Hoon Ko, M.S^2,†^, Nan Yeol Kim, M.D^1,†^, Do Wan Kim, M.D^3^, Jayun Cho, M.D^4^, Hongjin Shim, M.D^5^, Jin Goo Kim, M.S^6^, Ji Young Jang, M.D^7^, Kyung Won Kim, M.D^8^ and Jinseok Lee, Ph.D^2,*^

^1^Department of Trauma Surgery, Jeju Regional Trauma Center, Cheju Halla General Hospital, Jeju, Republic of Korea
^2^Department of Biomedical Engineering, Kyung Hee University, Yongin, Republic of Korea
^3^Department of Thoracic and Cardiovascular Surgery, Chonnam National University Hospital and Chonnam National University Medical School, Gwangju, Republic of Korea
^4^Department of Trauma Surgery, Gachon University Gil Medical Center, Republic of Korea
^5^Wonju Trauma Center, Yonsei University Wonju College of Medicine, Wonju, Republic Korea
^6^Trauma Center, Wonkwang University Hospital, Iksan, Republic of Korea
^7^Department of Surgery, National Health Insurance Service, Ilsan Hospital, Goyang, Republic of Korea
^8^Asan Medical Center, University of Ulsan College of Medicine, Seoul, Republic of Korea
^*^gonasago@khu.ac.kr
†these authors contributed equally to this work.

Supplementary Table 1. The age and gender information for each of four trauma centers

| Data sources | Age | | Gender | | | | | |
| --- | --- | --- | --- | --- | --- | --- | --- | --- |
|  | Survived | Deceased | Male | | | Female | | |
|  |  |  | Total | Survived | Deceased | Total | Survived | Deceased |
| WKUH | 59.97 ± 17.87 | 64.64 ± 16.52 | 2,734 | 2,597 | 137 | 1,578 | 1,538 | 40 |
| JNUH | 59.37 ± 17.73 | 59.86 ± 17.39 | 6,737 | 6,440 | 297 | 3,509 | 3,368 | 141 |
| WSCH | 59.25 ± 17.70 | 60.31 ± 17.58 | 9,226 | 8,976 | 250 | 4,780 | 4,666 | 114 |
| GUGH | 59.46 ± 17.81 | 60.04 ± 17.61 | 6,.037 | 5,855 | 182 | 3,161 | 3,053 | 108 |
| Total | 57.00 ± 17.80 | 64.65 ± 16.42 | 24,734 | 23,868 | 866 | 13,028 | 12,625 | 403 |

Supplementary Table 2. Full list of Region-46

The list is with a separated excel file.

Supplementary Table 3. Region-46 feature lists and the number of patients accordingly. Each patient may belong to multiple features

| No. | Organ feature | The number of patients | No. | Organ feature | The number of patients |
| --- | --- | --- | --- | --- | --- |
| 1 | skull | 3,019 | 24 | bronchus | 1 |
| 2 | head | 7,200 | 25 | thorax esophagus | 9 |
| 3 | cerebrum | 1,599 | 26 | thorax trachea | 5 |
| 4 | cerebrum epidural | 1,107 | 27 | diaphragm | 96 |
| 5 | cerebrum subdural | 4,607 | 28 | abdomen | 1,428 |
| 6 | intracerebral | 1,103 | 29 | liver | 1,192 |
| 7 | cerebellum | 39 | 30 | kidney | 392 |
| 8 | cerebellum epidural | 47 | 31 | abdomen vessel | 458 |
| 9 | cerebellum subdural | 74 | 32 | lumbar spine | 3,256 |
| 10 | intracerebellar | 36 | 33 | spleen | 591 |
| 11 | neck | 250 | 34 | stomach | 66 |
| 12 | cervical spine | 3,161 | 35 | omentum | 98 |
| 13 | neck vessel | 40 | 36 | duodenum | 60 |
| 14 | neck esophagus | 9 | 37 | pancreas | 155 |
| 15 | neck trachea | 15 | 38 | colon | 206 |
| 16 | face | 8,310 | 39 | small bowel | 355 |
| 17 | thorax | 4,763 | 40 | mesentery | 447 |
| 18 | thoracic spine | 1,933 | 41 | rectum | 23 |
| 19 | thoracic vessel | 153 | 42 | bladder | 75 |
| 20 | lung | 1,427 | 43 | upper extremity | 11,548 |
| 21 | rib | 6,147 | 44 | pelvis | 2,527 |
| 22 | heart | 93 | 45 | lower extremity | 12,280 |
| 23 | pericardium | 53 | 46 | external | 712 |

Supplementary Table 4. Comparison of cross-validation evaluation metrics (mean ± standard deviation)

|  | Cross-validation results | | | |
| --- | --- | --- | --- | --- |
|  | Sensitivity (%) | Specificity (%) | Accuracy (%) | **Balanced Accuracy (%)** |
| DNN(AIS) | 74.18 ± 10.04 | 87.11 ± 11.06 | 86.67 ± 10.36 | **80.64 ± 1.46** |
| DNN(Region-6) | 83.95 ± 5.24 | 82.98 ± 4.75 | 82.73 ± 4.42 | **83.32 ± 1.07** |
| DNN(Region-46) | 83.38 ± 5.83 | 84.99 ± 5.54 | 84.99 ± 5.19 | **84.61 ± 1.22** |

Supplementary Table 5. Testing data result comparison with LR, RF, SVM and DNN, each of which has the input features of AIS, Region-6 or Region-46 according to each of four trauma centers

| Model | Data sources | TN | FP | FN | TP | Sensitivity | Specificity | Accuracy | Balanced Accuracy |
| --- | --- | --- | --- | --- | --- | --- | --- | --- | --- |
| LR(AIS) | WKUH | 379 | 34 | 5 | 13 | 0.7222 | 0.9177 | 0.9095 | 0.8199 |
|  | JNUH | 808 | 173 | 14 | 30 | 0.6818 | 0.8236 | 0.8176 | 0.7527 |
|  | WSCH | 1,212 | 152 | 5 | 31 | 0.8611 | 0.8886 | 0.8879 | 0.8748 |
|  | GUGH | 801 | 90 | 8 | 21 | 0.7241 | 0.8990 | 0.8935 | 0.8116 |
|  | **Total** | **3,200** | **449** | **32** | **95** | **0.7480** | **0.8770** | **0.8726** | **0.8125** |
| RF(AIS) | WKUH | 330 | 83 | 4 | 14 | 0.7778 | 0.7990 | 0.7981 | 0.7884 |
|  | JNUH | 649 | 332 | 6 | 38 | 0.8636 | 0.6616 | 0.6702 | 0.7626 |
|  | WSCH | 1,083 | 281 | 5 | 31 | 0.8611 | 0.7940 | 0.7957 | 0.8275 |
|  | GUGH | 658 | 233 | 5 | 24 | 0.8276 | 0.7385 | 0.7413 | 0.7830 |
|  | **Total** | **2,720** | **929** | **20** | **107** | **0.8425** | **0.7454** | **0.7487** | **0.7940** |
| SVM(AIS) | WKUH | 353 | 60 | 3 | 15 | 0.8333 | 0.8547 | 0.8538 | 0.8440 |
|  | JNUH | 777 | 204 | 9 | 35 | 0.7955 | 0.7920 | 0.7922 | 0.7938 |
|  | WSCH | 1,151 | 213 | 2 | 34 | 0.9444 | 0.8438 | 0.8464 | 0.8941 |
|  | GUGH | 751 | 140 | 7 | 22 | 0.7586 | 0.8429 | 0.8402 | 0.8007 |
|  | **Total** | **3,032** | **617** | **21** | **106** | **0.8346** | **0.8309** | **0.8310** | **0.8328** |
| DNN(AIS) | WKUH | 383 | 30 | 4 | 14 | 0.7778 | 0.9274 | 0.9211 | 0.8526 |
|  | JNUH | 815 | 166 | 14 | 30 | 0.6818 | 0.8308 | 0.8244 | 0.7563 |
|  | WSCH | 1,229 | 135 | 6 | 30 | 0.8333 | 0.9010 | 0.8993 | 0.8672 |
|  | GUGH | 803 | 88 | 9 | 20 | 0.6897 | 0.9012 | 0.8946 | 0.7954 |
|  | **Total** | **3,230** | **419** | **33** | **94** | **0.7402** | **0.8852** | **0.8803** | **0.8127** |
| LR(Region-6) | WKUH | 359 | 54 | 2 | 16 | 0.8889 | 0.8692 | 0.8701 | 0.8791 |
|  | JNUH | 797 | 184 | 13 | 31 | 0.7045 | 0.8124 | 0.8078 | 0.7585 |
|  | WSCH | 1,160 | 204 | 3 | 33 | 0.9167 | 0.8504 | 0.8521 | 0.8836 |
|  | GUGH | 743 | 148 | 7 | 22 | 0.7586 | 0.8339 | 0.8315 | 0.7963 |
|  | **Total** | **3,059** | **590** | **25** | **102** | **0.8031** | **0.8383** | **0.8371** | **0.8207** |
| RF(Region-6) | WKUH | 365 | 48 | 2 | 16 | 0.8889 | 0.8838 | 0.8840 | 0.8863 |
|  | JNUH | 785 | 196 | 10 | 34 | 0.7727 | 0.8002 | 0.7990 | 0.7865 |
|  | WSCH | 1,181 | 183 | 5 | 31 | 0.8611 | 0.8658 | 0.8657 | 0.8635 |
|  | GUGH | 759 | 132 | 7 | 22 | 0.7586 | 0.8519 | 0.8489 | 0.8052 |
|  | **Total** | **3,090** | **559** | **24** | **103** | **0.8110** | **0.8468** | **0.8456** | **0.8289** |
| SVM(Region-6) | WKUH | 354 | 59 | 3 | 15 | 0.8333 | 0.8571 | 0.8561 | 0.8452 |
|  | JNUH | 780 | 201 | 8 | 36 | 0.8182 | 0.7951 | 0.7961 | 0.8066 |
|  | WSCH | 1,143 | 221 | 4 | 32 | 0.8889 | 0.8380 | 0.8393 | 0.8634 |
|  | GUGH | 732 | 159 | 8 | 21 | 0.7241 | 0.8215 | 0.8185 | 0.7728 |
|  | **Total** | **3,009** | **640** | **23** | **104** | **0.8189** | **0.8246** | **0.8244** | **0.8218** |
| DNN(Region-6) | WKUH | 363 | 50 | 2 | 16 | 0.8889 | 0.8789 | 0.8794 | 0.8839 |
|  | JNUH | 779 | 202 | 9 | 35 | 0.7955 | 0.7941 | 0.7941 | 0.7948 |
|  | WSCH | 1,150 | 214 | 3 | 33 | 0.9167 | 0.8431 | 0.8450 | 0.8799 |
|  | GUGH | 736 | 155 | 6 | 23 | 0.7931 | 0.8260 | 0.8250 | 0.8096 |
|  | **Total** | **3,028** | **621** | **20** | **107** | **0.8425** | **0.8298** | **0.8302** | **0.8362** |
| LR(Region-46) | WKUH | 362 | 51 | 2 | 16 | 0.8889 | 0.8765 | 0.8770 | 0.8827 |
|  | JNUH | 816 | 165 | 11 | 33 | 0.7500 | 0.8318 | 0.8283 | 0.7909 |
|  | WSCH | 1,171 | 193 | 3 | 33 | 0.9167 | 0.8585 | 0.8600 | 0.8876 |
|  | GUGH | 760 | 131 | 8 | 21 | 0.7241 | 0.8530 | 0.8489 | 0.7886 |
|  | **Total** | **3,109** | **540** | **24** | **103** | **0.8110** | **0.8520** | **0.8506** | **0.8315** |
| RF(Region-6) | WKUH | 361 | 52 | 2 | 16 | 0.8889 | 0.8741 | 0.8747 | 0.8815 |
|  | JNUH | 777 | 204 | 10 | 34 | 0.7727 | 0.7920 | 0.7912 | 0.7824 |
|  | WSCH | 1,168 | 196 | 5 | 31 | 0.8611 | 0.8563 | 0.8564 | 0.8587 |
|  | GUGH | 748 | 143 | 6 | 23 | 0.7931 | 0.8395 | 0.8380 | 0.8163 |
|  | **Total** | **3,054** | **595** | **23** | **104** | **0.8189** | **0.8369** | **0.8363** | **0.8279** |
| SVM(Region-46) | WKUH | 365 | 48 | 3 | 15 | 0.8333 | 0.8838 | 0.8817 | 0.8586 |
|  | JNUH | 788 | 193 | 8 | 36 | 0.8182 | 0.8033 | 0.8039 | 0.8107 |
|  | WSCH | 1,173 | 191 | 4 | 32 | 0.8889 | 0.8600 | 0.8607 | 0.8744 |
|  | GUGH | 765 | 126 | 8 | 21 | 0.7241 | 0.8586 | 0.8543 | 0.7914 |
|  | **Total** | **3,091** | **558** | **23** | **104** | **0.8189** | **0.8471** | **0.8461** | **0.8330** |
| DNN(Region-46) | WKUH | 361 | 52 | 2 | 16 | 0.8889 | 0.8741 | 0.8747 | 0.8815 |
|  | JNUH | 814 | 167 | 9 | 35 | 0.7955 | 0.8298 | 0.8283 | 0.8126 |
|  | WSCH | 1,202 | 162 | 3 | 33 | 0.9167 | 0.8812 | 0.8821 | 0.8989 |
|  | GUGH | 784 | 107 | 7 | 22 | 0.7586 | 0.8799 | 0.8761 | 0.8193 |
|  | **Total** | **3,161** | **488** | **21** | **106** | **0.8346** | **0.8663** | **0.8652** | **0.8505** |
